# Nodular fasciitis of the external ear masquerading as pleomorphic adenoma: A potential diagnostic pitfall in fine needle aspiration cytology

**DOI:** 10.4103/1742-6413.44242

**Published:** 2008-11-26

**Authors:** Deepali Jain, Nita Khurana, Shyama Jain

**Affiliations:** Department of Pathology, Maulana Azad Medical College, New Delhi, India

**Keywords:** Cytology, nodular fasciitis, pleomorphic adenoma, post-aural region

## Abstract

**Background:**

Nodular fasciitis (NF) is a benign myofibroblastic proliferation in soft tissue. The most common sites are extremities, followed by the trunk and head and neck region. It is infrequently seen in the post-auricular region of pinna.

**Case presentation:**

We present here an interesting case of a young male who had a swelling in the post-auricular region; on cytology, it was diagnosed as pleomorphic adenoma; however, biopsy revealed characteristic morphology of NF.

**Conclusion:**

The case highlights the potential pitfall of cytology in diagnosing NF, especially because of unusual site and morphologic overlap.

Nodular fasciitis (NF) was originally described by Konwaler *et al.* and is synonymous with pseudosarcomatous fasciitis.[1] These lesions are mostly located in the soft tissues of the forearm, followed by trunk and head and neck.[2] It is extremely rare in the external ear. Although there are a few histological reports on NF involving the post-auricular region, published cytological literature is too scanty, and NF may be confused with other neoplastic lesions of the salivary gland.[3,4]

We report here an interesting case of an adolescent boy who presented with a swelling in the post-auricular region; and on cytology, it was diagnosed as pleomorphic adenoma (PA); however, the histological diagnosis of classical NF was a surprise. The case is presented to highlight the diagnostic pitfall of cytology.

## Case presentation

A 16-year-old adolescent boy presented with a swelling in the post-auricular region, slowly increasing in size for the past 6 weeks. On examination, the swelling was nontender, nonfluctuant, smooth, soft to firm with well-defined margins and measuring 1.5 cm in diameter. Clinical diagnosis of sebaceous cyst was considered, and the patient was referred for fine needle aspiration cytology (FNAC).

## Cytological findings

**Figure 1:**
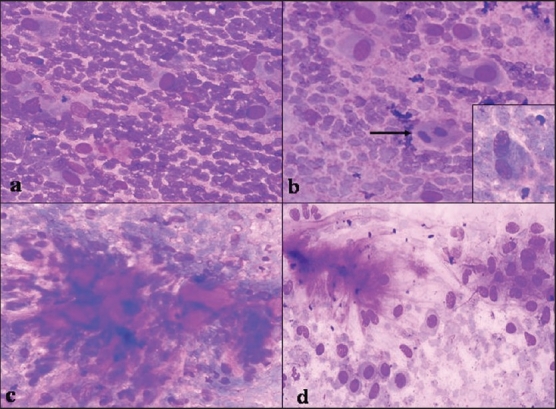
FNA smears — a) dissociated plasmacytoid cells with central-to-eccentric nuclei, and fragile cytoplasm; b) plasmacytoid cells, typical mitotis (arrow), and binucleated ganglionlike cells with prominent nucleoli (inset); c) spherical magenta-colored stromal fragments surrounded by spindle-to-oval cells; d) FNA from a known case of pleomorphic adenoma, for comparison: plasmacytoid cells with well-demarcated cytoplasmic borders, indistinct nucleoli, fibrillary stroma. Giemsa stain ×300

FNA smears showed moderate cellularity comprising of predominantly dissociated round-to-plasmacytoid cells with central-to-eccentric nuclei, occasional binucleated cells, and few spindle cells. A fair number of spherical and linear deep magenta–colored stromal fragments surrounded by similar cells were also seen. The background was pale mucoid. There was no pleomorphism and necrosis; however, occasional typical mitosis was noticed [Figures 1]. A cytological diagnosis of PA was suggested, although a possibility of skin adnexal tumor was also considered as the swelling was involving the external ear.

## Histological findings

**Figure 2:**
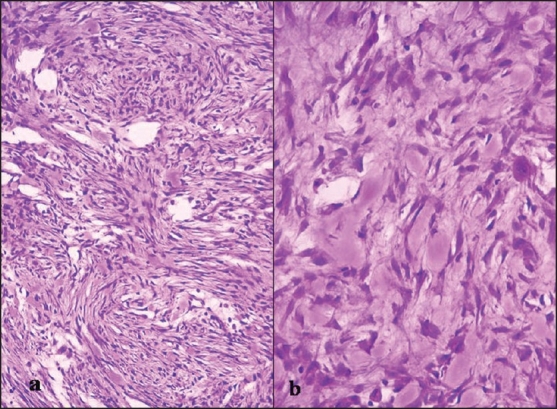
Section from resected nodule — a) spindle-to-oval cells in a storiform pattern (H and E ×150); b) spindle-to-plasmacytoid cells with interspersed ganglionlike cells (arrow) present in collagenous stroma (H and E ×300)

Subsequently, total excision of the mass was performed for histological correlation. Gross examination showed a well-circumscribed, unencapsulated gray-white soft tissue mass measuring 1.5×1 cm in size. Histologically, the lesion comprised of proliferating but uniform-appearing plump fibroblasts arranged loosely in short fascicles, bundles, and vague storiform patterns. The background stroma was variably collagenous. Few mitotic figures and extravasated red blood cells were also noted [Figure 2]. In view of these findings, a histological diagnosis of nodular fasciitis of external ear was established.

## Immunohistochemical studies

A panel of immunohistochemical (IHC) markers for cytokeratin (CK), epithelial membrane antigen (EMA), S-100, smooth muscle actin (SMA), and vimentin (Vim) was performed by Avidin-Biotin complex (ABC) method. On IHC study, CK, EMA, and S-100 were negative, and SMA and Vim were focally expressed, thus confirming the diagnosis of NF.

FNA smears reviewed after histological diagnosis of NF for the overlapping features of NF and PA [Table 1, Figure 1] were found consistent with the final diagnosis of NF.

**Table 1:** Differentiating features of pleomorphic adenoma and nodular fasciitis in fine needle aspiration cytology

*Cytological features*	*Pleomorphic adenoma*	*Nodular fasciitis*	*Present case*
Cellularity	Variable, usually high	High	High
Arrangement	Dissociated, clusters	Dissociated, sheets, fascicles, storiform	Dissociated occasional cluster
Cell shape			
Plasmacytoid cells	+	+	+
Triangular, polyhedral cells	−	+	*+
Spindle cells	*	*	*
Epithelial cells	+	−	−
Cytoplasm			
		Abundant, purple	Moderate, purple
Amount, color	Moderate, purple	Fuzzy	* Fuzzy
Cytoplasmic borders	Well demarcated	Frequent	* Frequent
Delicate plasmatic processes in ganglion like cells	Absent	May be present (in ganglion like cells)	*Absent
Pink granules	Absent		
Nucleus			
Position	Usually eccentric	Usually eccentric	Usually eccentric
Chromatin	Uniform chromatin	Uniform chromatin	Uniform chromatin
Pleomorphism	Little	Usually marked	Moderate
Bi/multi nucleation	Rare	Frequent	Frequent
Nucleoli	Indistinct	Large, prominent	Large, prominent
Mitosis	Absent (unless malignant)	Frequent (typical)	−(**)
Background			
Mucomyxomatous	Usually+	Usually+	+
Fibrillary	Usually+	Usually+	+
Metaplastic (chondroid, osseous)	Usually+	−	−
Inflammatory cells	−	Variable	−

* On review of FNA smear after knowing the histological diagnosis

** Present in biopsy

## Discussion

Nodular fasciitis is a relatively common soft tissue tumor; it occurs more frequently than most other tumors or tumorlike lesions of fibrous tissue that develop in adults.[1] Although several mechanisms are proposed including reactive or inflammatory process, the pathogenesis of NF is still unknown. Antecedent trauma has been suggested as an inciting factor.[5] We did not find such history in our patient. Although the most common site is upper extremity, it may arise from the subcutaneous tissue, muscle, or fascia at any location in the body.[2] It is rare in the external ear and hence is usually not considered in the differential diagnosis (D/D) of lesions in the pinna.[6] Interestingly, cases in the head and neck region often involve dermal tissue[7] ; the present case also demonstrated dermal NF. Thompson *et al.* studied clinico-pathological features in the largest series of 50 cases of NF in the region of external ear, including 28 dermal variants. In the auricular area, it occurs most often in young patients.[6] Usually, the lesion is smooth and may present sometimes with superficial ulceration also. Since our patient had a mass lesion in the post-auricular region, it was diagnosed as benign mixed tumor of the salivary gland or skin based on cytomorphological features.

Dahl and Ackerman first documented the correlation between FNAC and histologically confirmed cases of NF.[8] Aydin *et al.* further supported its cytological diagnosis.[9] Morphologically on both cytology and histology, NF may be easily mistaken for malignant neoplasm of spindle cells, tumors of salivary gland and skin with similar morphology, leading to unnecessary surgical treatment. The wide spectrum of morphological patterns of PA often presents a potential for errors in cytological interpretations.[10] The common morphological features shared by both PA and NF are spindle and plasmacytoid cells with central-to-eccentric nuclei, clumps of intercellular stromal material, and myxoid background. Mitotic figures are frequent in most cases of NF but sparse in cases of PA.[3] The present case was misdiagnosed as PA based on cytomorphological overlap and occurrence at an unusual site. The smear in the present case was carefully compared and evaluated after the histological diagnosis of NF, for detailed morphological features of classical PA and NF [Table 1]. On review of the smears, an important feature was noticed — that the spindle and plasmacytoid cells had fuzzy cytoplasmic borders, one-to-multiple fragile cytoplasmic processes, and prominent nucleoli similar to ganglionlike cells of NF [Figures 1a-1c]. In contrast, cells in PA show well-demarcated cytoplasmic borders and indistinct nucleoli [Figure 1d], in addition to other features which are helpful in differentiating NF from PA [Table 1].

Spontaneous regression is known to occur in NF even without excision. Local recurrences are more frequent in these cases because of the difficulty in obtaining complete surgical excision around the ear; rarely, frequent recurrences may transform the lesion to fibrosarcoma.[6]

To conclude, preoperative cytological diagnosis of NF is important to avoid unnecessary surgery in most cases. Although site and overlapping morphology can mislead the cytologist, leading to a diagnostic pitfall, a careful evaluation of certain morphological features may help in making a definitive diagnosis of NF.

## Competing interests

Authors do not have competing interests in this case.

## Authors' contributions

DJ, assistant professor, collected the literature and helped in drafting of the manuscript.

NK, professor, helped in the histological evaluation of the case.

SJ, professor, helped in the final revision and approval of the version for publication.

All authors read and approved the final manuscript.
